# Effect of gut microbiota changes on cytokines IL-10 and IL-17 levels in liver transplantation patients

**DOI:** 10.1186/s12879-025-10466-9

**Published:** 2025-01-30

**Authors:** Mohamed Abdel-Raoof Fouda, Mohamed Abdel-Wahhab, Ahmed Esmail Abdelkader, Mohsen El-Sayd Ibrahim, Taher Abozeid Elsheikh, Hisham Mohammad Aldeweik, Nora Elfeky

**Affiliations:** 1 Microbiology, Faculty of Medicine, Mansoura University, Mansoura, Egypt; 2 General Surgery, Faculty of Medicine, Mansoura University, Mansoura, Egypt; 3 Faculty of Science, Port Said University, Port Said, Egypt; 4 Microbiology Lab., Environmental Monitoring Administration, Ministry of Health, Mansoura, Egypt; 5 Botany and Microbiology Department, Faculty of Science, Menoufia University, Shebeen El-Kom, Egypt

**Keywords:** Liver transplantation, Gut microbiota, Cytokine, IL-10, IL-17, Nosocomial infection

## Abstract

**Background:**

Liver transplantation (LT) is a critical intervention for individuals with end-stage liver disease; yet, post-transplant problems, especially infections, graft rejection, and chronic liver disease, are often linked to systemic inflammation. Cytokines, small signaling molecules, significantly influence immune responses during and post-liver transplantation. Nonetheless, the intricate relationships among cytokines, immune responses, and the gut microbiota, especially gut dysbiosis, are still inadequately comprehended. Thus, this study aims to identify the gut microbiota (GM) and determine their relationship to cytokines (IL-17 and IL-10) in LT patients, due to their importance in enhancing the recovery rate.

**Result:**

The research included 31 liver transplant (LT) patients from the Gastroenterology Surgical Center at Mansoura University, resulting in the collection of 174 stool and blood samples from all participants. Fourteen bacterial species have been identified in samples collected at three intervals: one week before, one week post, and two weeks post LT. A change in gut microbiota composition was noted, characterized by a rise in potentially pathogenic bacteria such as Enterococci and Enterobacteriaceae (including *Escherichia coli* and *Klebsiella*) and a reduction in beneficial bacteria such as Bacteroidetes and Firmicutes. The examination of patient demographic and clinical data revealed no significant correlations between sex, age, or diagnostic categories and gut microbiota composition. The findings of the Multivariate Analysis of Variance (MANOVA) indicated a substantial effect of gut microbiota composition on cytokine levels (IL-10 and IL-17), with all tests producing p-values of 0.001. The assessment of cytokine levels indicated fluctuating variations at several time points following surgery. IL-10 levels in the GM groups exhibited a statistically significant elevation during the second week post-surgery (*p* = 0.036), suggesting a potential recovery-related anti-inflammatory response. In contrast, IL-17 levels rose in the NI group over time, indicating a transition to a pro-inflammatory condition.

**Conclusion:**

This study emphasizes the pivotal role of the gut microbiota in regulating immune responses following transplantation.

## Introduction

Liver transplantation (LT) is a critical intervention for individuals with end-stage liver disease, offering a potential remedy for liver failure, cirrhosis, and other chronic hepatic disorders. Despite significant advancements in surgical techniques and immunosuppressive therapies, liver transplant recipients still encounter a great risk of complications, such as graft rejection, infections, and chronic allograft dysfunction. Recent studies demonstrate that the gut microbiome, the complex collection of microorganisms within the gastrointestinal tract, profoundly impacts immune responses and determines post-transplant results [1–3]. The human gastrointestinal tract (GIT) has a complex and diverse assemblage of microorganisms known as gut microbes (GM). The gut microbiota significantly influences the immune system by its interactions with the host’s immune cells, particularly those involved in inflammation and immunological tolerance [4]. Both LT and end-stage liver disease are frequently linked to modifications in the composition of the gastrointestinal microbiota (GM), referred to as gut dysbiosis, which is induced by antimicrobial therapy, medical interventions, anatomical alterations from medical procedures, biliary complications, and the administration of immunosuppressive agents [5]. Recent studies demonstrate that dysbiosis in liver transplant patients is associated with an elevation of pathogenic organisms and a reduction of helpful bacteria, resulting in altered immune responses that affect graft survival and patient outcomes [6, 7]. A recent stool analysis indicates that Bacteroidetes, Firmicutes, Proteobacteria, and Actinobacteria are the main bacterial taxa in the gastrointestinal tract (GIT) [8]. Proteobacteria is a significant group of gram-negative bacteria that includes several pathogenic species, such as *Pseudomonas*, *E. coli*, and *Klebsiella* spp [8].

The probability of illness among liver transplant recipients fluctuates over time [9]. The difficulties encountered in the early post-transplant phase frequently pertain to complications related to the transplant operation and the emergence of nosocomial infections (NIs) [10]. Opportunistic infections are more likely to arise within the first year post-transplant due to heightened immunosuppression. Nonetheless, the incidence of opportunistic infections decreases when the level of immunosuppression lessens after post-donation [11]. Transplant recipients continue to be vulnerable to nosocomial infections, and those with extended allograft failure or recurrent cholestatic liver disease may experience recurrent cholangitis [12]. Nosocomial infection (NI) is an ailment acquired within a healthcare setting. Contamination may occur in diverse clinical environments, such as clinics, nursing homes, rehabilitation centers, outpatient facilities, and analogous healthcare institutions [13].

Interleukin-10 (IL-10) and interleukin-17 (IL-17) are significant immune mediators affected by changes in the gut microbiota, due to their critical roles in immune regulation IL-10, an anti-inflammatory cytokine, is essential for maintaining immunological homeostasis, particularly in alleviating overactive immune responses that could lead to graft rejection [14, 15]. In contrast, IL-17, a pro-inflammatory cytokine, is involved in the recruitment and activation of immune cells during inflammation and infection [16, 17]. Dysregulation of these cytokines can lead to imbalanced immune responses, resulting in graft rejection and related complications following liver transplantation [18]. The immune system continuously interacts with commensal bacteria. Mounting data suggests that commensal bacteria significantly influence the immunological tests employed in organ transplantation. Nonetheless, the inquiry into the relationship between transplant resistance and commensals is presently in its first stages. Research indicates that alterations in gut microbiota can significantly influence the production of IL-10 and IL-17. Studies employing animal models have shown that changes in microbial communities can affect the expression of these cytokines, hence influencing the balance between immune tolerance and inflammation [4]. Clinical investigations in humans have demonstrated that changes in gut microbiota composition correlate with fluctuations in cytokine levels, which in turn affect post-transplant recovery and the likelihood of rejection [19, 20]. The specific mechanisms linking gut dysbiosis to cytokine dysregulation in liver transplant recipients remain poorly elucidated, warranting more investigation. This study aims to examine the effects of changes in the gut microbiome on IL-10 and IL-17 levels in liver transplant recipients. By improving our understanding of the gut-liver axis and its role in immune modulation, we aim to identify potential therapeutic targets to enhance the effectiveness of liver transplantation.

## Materials and methods

### LT patients

The study population included 31 LT patients, in the period from March 2021 to March 2023. All patients were recruited from Gastroenterology Surgical Center (GEC), Mansoura University, Mansoura, Egypt. All individuals provided informed consent and were fully informed about the diagnostic methods involved and the nature of the condition. The study procedure followed the Helsinki Declaration 2013 ethical guidelines and was approved by the research ethics council of Faculty of Medicine, Mansoura University (IRB: MDP.23.08.131.R1). Before taking part in the study, all patients provided written informed consent.

### Dietary management

Pre- and post-liver transplantation, the diet is adjusted to promote optimal liver function, prevent excessive weight gain, and support overall health. Patients are instructed to consume at least five servings of fruits and vegetables daily, and to avoid fish for the first six months following transplantation. Whole grains, such as whole-grain breads and cereals, are encouraged to provide necessary nutrients and fiber. Additionally, a sufficient intake of fiber is recommended throughout the diet. Patients are advised to drink low-fat milk or other low-fat dairy products, and to maintain a low-salt and low-fat diet. Alcohol consumption is strictly prohibited, and patients are instructed to stay hydrated by drinking adequate amounts of water and other fluids daily. Grapefruit and grapefruit juice are also avoided due to their potential interaction with immunosuppressive medications, which may alter their effectiveness.

### Immunosuppression protocol

A steroid-free immunosuppression protocol is adopted for all patients in this study. Induction immunosuppression consists of administering Basiliximab on Day 0 and Day 4. Additionally, a single intravenous dose of methylprednisolone (500 mg) is given. Tacrolimus (Prograf) is the primary immunosuppressive agent, unless patients experience severe kidney injury, in which case the initiation of tacrolimus is delayed until Day 4. During the first 14 days post-transplantation, the prescribed tacrolimus dose is 0.01 mg/kg every 12 h. To prevent infections, all patients post-liver transplantation are administered a combination of antibiotics, including Tazocin, Tienam, Meropenem, Maxipime, and Vancomycin.

### Sample collection

The stool and blood samples were collected from the 31 patients at three separate time periods: one week before LT (*n* = 31) (1WB), one week after LT (*n* = 28) (1WA) and two weeks after LT (*n* = 28) (2WA). A total of 174 samples were taken from all patients, divided between stool (*n* = 87) and blood (*n* = 87). Three patients’ data were missing one week after and two week after LT surgery because exit immediately before the surgery based on vision of doctors.

### Detection of GM

Stool samples were divided into two equal subsamples for aerobic and anaerobic cultivation. For the aerobic specimens, the samples were transported in 5 ml of sterile Amies transport media [21] (OXOID- England) at 4 °C within 2 h. These samples were initially cultured in Brucella broth medium [22] (OXOID- England) as an enrichment medium, followed by subculturing on Columbia blood agar base medium [23] for further bacterial growth. For the anaerobic specimens, samples were collected directly onsite in plastic containers and placed in a vacuum box. Anaerobic gas generator sachets were added to the vacuum box immediately after collection. Within 30 min, the box was transferred to an anaerobic chamber, where it was opened, and the specimens were cultured on Columbia Chocolate agar base medium [24] (OXOID- England)to promote the growth of anaerobic bacteria. The isolates were subsequently purified on nutrient agar [24]. The pure bacterial isolates were identified using the VITEK 2 Compact 15 system (BioMérieux, France).

### Estimation of cytokines IL-10 and IL-17 levels in blood samples

Blood samples were collected from the patients’ peripheral veins following an overnight fasting period. The samples were transferred into sterile tubes and allowed to stand undisturbed for 30 min to facilitate clotting. Afterward, the serum was separated by centrifugation for 15 min at 3000 rpm. The serum was carefully extracted using an automated pipette and transferred into aseptic tubes for storage at -20 °C.The blood samples were classified based on the results of stool cultures, and the occurrence of nosocomial infections. Eight patients developed infections in the first week after liver transplantation (LT), and four of these patients recovered by the second week of post-surgery. The remaining four patients continued to have nosocomial infections into the second week following LT. None of the patients exhibited bacterial infections in the week prior to LT.

Cytokine levels of IL-10 and IL-17 were measured using the enzyme-linked immunosorbent assay (ELISA) method, utilizing ELISA kits from Bio-Techne (UK). The sensitivity range for IL-10 detection was 15.5–1000 pg/mL, and for IL-17, the sensitivity range was 93.75–6000 pg/mL [25].

### Statistical analysis

The Shapiro-Wilk test determined data normality for all groups. This test determined if each group’s data fitted a normal distribution. The data was normal if each group’s p-value was larger than 0.05, and non-normal if it was less than 0.05. Different statistical tests were used to detect the significant differences between groups. All statistical analyses were performed using The Statistical Package for the Social Sciences (SPSS) programming application 15.0 (SPSS Inc., Chicago, IL), with significance set at *p* < 0.05.

## Results

### Demographic data of LT patients

Thirty-one LT patients classified by sex, age, diagnosis, and gut microbiota status (Table 1). A Mann-Whitney U test was employed to evaluate differences in gut microbiota between male and female patients. Among the 31 patients, 25 (80.6%) were male and 6 (19.4%) were female, with a p-value of 0.648, suggesting no significant variation in gut microbiota between genders.

The predominant age group of patients was 40 to 50 years, comprising 48.4% of the total. A Spearman’s rho correlation was conducted to assess the relationship between age and gut microbiota, yielding a p-value of 0.593. This indicates a lack of significant correlation between age and gut microbiota in the sample. Of the patients diagnosed, 12 (38.7%) had cirrhosis, 9 (29.0%) had hepatocellular carcinoma (HCC), 3 (9.7%) had Budd-Chiari Syndrome (BCS), 6 (19.4%) had autoimmune hepatitis (AIH), and 1 (3.2%) had liver cell failure (LCF). A p-value of 0.743 signifies no significant alterations in gut microbiota relative to diagnosis. The distribution of gut microbiota conditions among patients was as follows: 74.1% displayed normal gut microbiota (NGM), 12.9% indicated nosocomial infection (NI) till the end of investigation, and 12.9% had recovered from nosocomial infection (RNI). The statistical analysis indicated no significant correlations or variances in gut microbiota associated with sex, age, or diagnosis within the study population.

**Table 1:** Patient data, percentages, and corresponding statistical test results

Variable	Category	*N* (%)	Statistical Test	*P*-Value
Sex	Male	25 (80.6%)	Mann-Whitney U	0.648
	Female	6.0 (19.4%)		
Age	< 30 years	3.0 (9.7%)	Spearman’s rho	0.593
	> 30–40 years	4.0 (12.9%)		
	> 40–50 years	6.0 (19.3%)		
	> 50–60 years	15 (48.4%)		
	> 60 years	3.0 (9.7%)		
Diagnosis	Cirrhosis	12 (38.7%)	Kruskal-Wallis H	0.743
	Hepatocellular Carcinoma (HCC)	9.0 (29.0%)		
	Budd-Chiari Syndrome (BCS)	3.0 (9.7%)		
	Autoimmune Hepatitis (AIH)	6.0 (19.4%)		
	Liver Cell Failure (LCF)	1.0 (3.2%)		
Gut Microbiota	NGM (Normal)	23 (74.1%)		
	NI (Nosocomial Infection)	4.0 (12.9%)		
	RNI (Recovered NI)	4.0 (12.9%)		

### Detection of GM in LT patients

The objective of this study was to identify the composition of the GM and changes in their composition in patients who had received a liver transplant. A total of 87 fecal specimens obtained from 31 patients with LT at three distinct time points were utilized for the isolation and identification of GM. The samples were categorized into three distinct groups and organized according to the time period preceding and following the LT surgery.

**Fig. 1:**
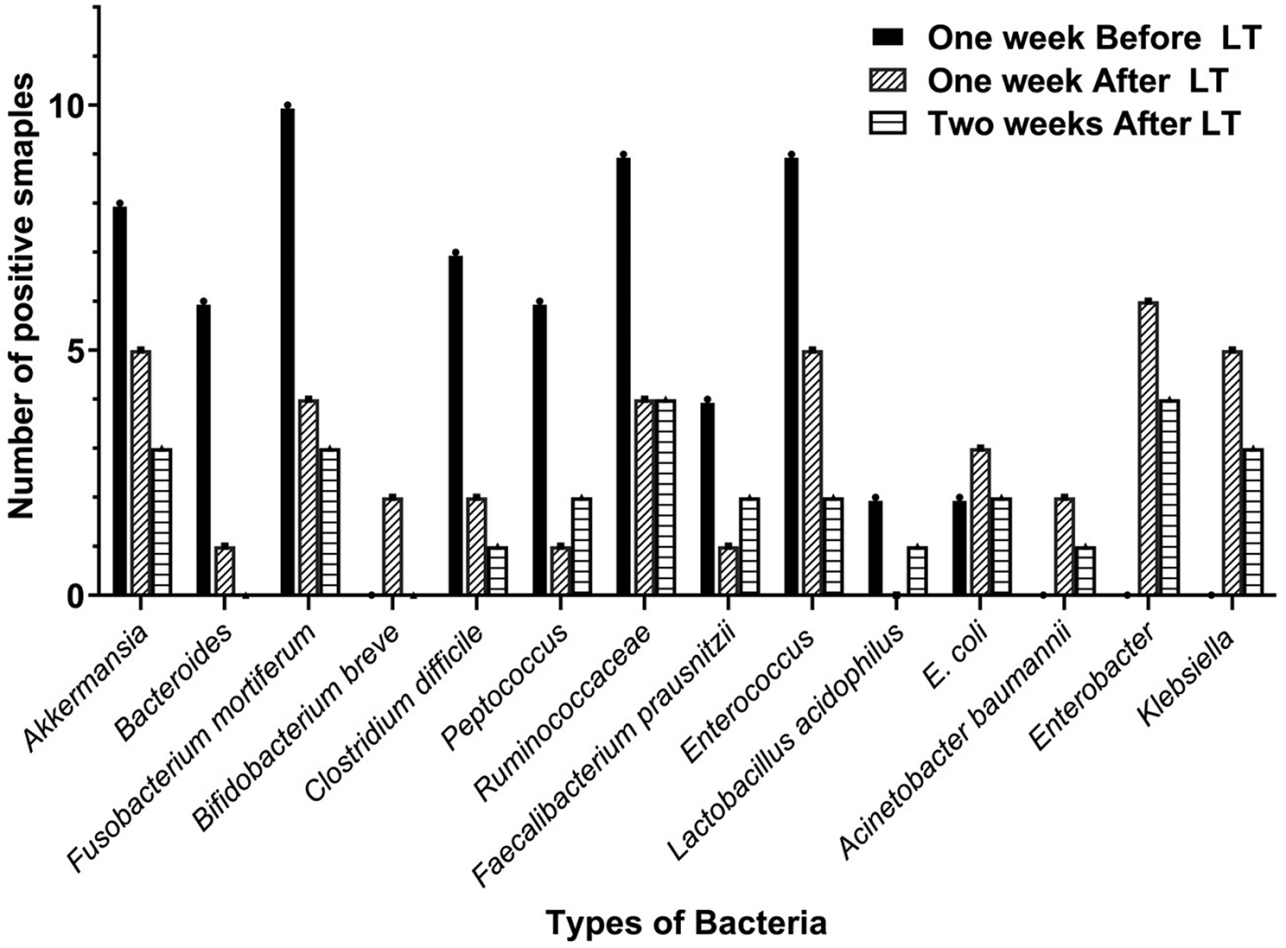
Isolated bacteria from stool cultures of LT patients

A total of 132 bacterial isolates one week before, after, and two weeks after LT surgery were identified (Fig. 1). In the week before LT surgery (*n* = 63), the most dominant bacterial isolates were *Fusobacterium mortiferum* 10 (15.9%), *Enterococcus* 9 (14.3%), *Ruminococcaceae* 9 (14.3%), *Akkermansia* 8 (12.7%), *Clostridium* 7 (11.1%), *Bacteroides* 6 (9.5%), *Peptococcus* 6 (9.5%), *Faecalibacterium prausnitzii* 4 (6.3%), *Lactobacillus*2(3.2%), and *E. coli* 2 (3.2%). The distribution of the dominating bacterial isolates were changed among the after one week specimens (*n* = 41) to be *Enterobacter aerogenes* 3 (7.3%), *Enterobacter cloaecae* 2 (4.9%), *Enterobacter hormaechei* 1 (2.4%), *K. pneumoniae* 3 (7.3%), *K. oxytoca* 2 (4.9%), *Acinetobacter baumannii* 2 (4.9%), *Akkermansia* 5(12.2%), *E. coli* 3 (7.3%), *B. melaninogenicus* 1 (2.4%), *Enterococcus faecalis* 2 (4.9%), *Enterococcus gallinarum* 1 (2.4%), *Enterococcus faecium* 2 (4.9%) *Fusobacterium mortiferum* 4 (9.8%), *Bifidobacterium breve* 2 (4.9%), *Clostridium* 2 (4.9%), *Ruminococcaceae* 4 (9.8%), *Peptococcus* 1 (2.4%), and *Faecalibacterium prausnitzii* 1 (2.4%). Also, the composition of the isolated bacteria from patients after two weeks LT surgery (*n* = 28) changed to be *Enterobacter* 4 (14.2%), *Ruminococcaceae* 4 (14.2%), *Akkermansia* 3 (10.7%), *Fusobacterium mortiferum* 3 (10.7%), *Klebsiella* 3 (10.7%), *Peptococcus* 2 (7.2%), *Faecalibacterium prausnitzii* 2 (7.2%), *Enterococcus* 2 (7.2%), *E. coli* 2 (7.2%), *Clostridium* 1 (3.6%), *Lactobacillus* 1 (3.6%), and *Acinetobacter baumannii* 1 (3.6%).

Changes in the composition of the GM are commonly linked to changed anatomy following surgery, antibiotic medication, and immunological suppression. The incidence of nosocomial infections in patients was as follows: cirrhosis 4 (50.0%), hepatocellular carcinoma (HCC) 2 (25.0%) and Autoimmune hepatitis (AIH) 2 (25.0%). Eight patients were diagnosed with acquired nosocomial infections (NIs) one week after undergoing liver transplantation (LT). The diagnosis was consistent with the internationally recognized criteria for nosocomial infections as outlined by the Centers for Disease Control and Prevention (CDC) [26]. The diagnostic criteria included the presence of clinical symptoms (e.g., fever, abdominal pain, or changes in vital signs), the onset of infection (no patients exhibited symptoms of infection at the time of hospitalization), the timing of infection relative to hospitalization (symptoms developed more than 72 h after admission), and microbiological evidence from stool cultures. The stool cultures identified pathogens from the Enterococcaceae and Enterobacteriaceae families, specifically *Escherichia coli* and *Klebsiella* species, which confirmed the occurrence of infection consistent with the defined nosocomial etiology. Sensitivity tests were conducted, and the patients received the appropriate antibiotics. Four cases of nosocomial infections were recovered by the second week following liver transplantation (LT) (RNI). However, the remaining four cases persisted with nosocomial infections and were identified as being caused by multi-drug-resistant bacteria by the second week post-LT (NI).

### Estimation of cytokines IL-10 and IL-17 levels in the blood samples

The 87 blood samples were classified into three groups: the first group consisted of LT patients have normal GM at the time of sample collection (NGM), the second group consisted of LT patients diagnosed with nosocomial infection till the end of investigation (NI), and the third group consisted of LT patients who had recovered from nosocomial infection (RNI). Cytokine concentrations were measured in the supernatants of one week before (1WB), one week after (1WA) and tow week after (2WA) of whole blood cultures in vitro. The levels of IL-10 and IL-17 in the blood samples of LT patients were estimated for all samples. The Multivariate Analysis of Variance (MANOVA) was conducted to detect the effect of GM and time factors on cytokine levels (Table 2). The results indicated that the various time points evaluated in the study did not exhibit a statistically significant impact on cytokine levels (IL-10 and IL-17) among the groups (p-values = 0.197). This indicates that temporal changes between time points did not significantly affect cytokine concentrations. Conversely, gut microbiota distribution (including NGM, NI, and RNI) shows a significant impact on cytokine levels (*p* = 0.001). This indicates that the makeup and state of the gut microbiota significantly influence immune response indicators.

**Table 2:** Multivariate analysis of variance (MANOVA) results for the effect of time and gut microbiota on cytokine levels (IL-10 and IL-17)

Effect	Test	F-Value	Hypothesis df	Error df	*P*-Value
Time	Pillai’s Trace	1.658	2	79	0.197
	Wilks’ Lambda	1.658	2	79	0.197
	Hotelling’s Trace	1.658	2	79	0.197
	Roy’s Largest Root	1.658	2	79	0.197
GM	Pillai’s Trace	5.105	4	160	0.001
	Wilks’ Lambda	5.102	4	158	0.001
	Hotelling’s Trace	5.098	4	156	0.001
	Roy’s Largest Root	7.597	2	80	0.001

Table 3 presents the estimated values of IL-10 and IL-17 in LT patients throughout three time periods for the various groups (NGM, NI, and RNI). The evaluation of cytokine levels revealed variable fluctuations at multiple time intervals post-surgery. No significant variations in IL-10 levels were detected between the groups during 1WB and 1WA, with p-values surpassing the 0.05 threshold (*p* = 0.350 and *p* = 0.225, respectively). During 2WA, a significant difference was detected between the NI and control group (NGM), with NI exhibiting reduced IL-10 levels (203 ± 115 pg/ml) in contrast to NGM (459 ± 225 pg/ml, *p* = 0.030). The IL-10 levels for RNI during 2WA (265 ± 118 pg/ml) neared statistical significance when compared to NGM (*p* = 0.091), suggesting a potential recovery-associated anti-inflammatory response.

**Table 3:** Estimated values of the cytokines IL-10 and IL-17 in the LT patients throughout three time periods: 1WB, 1WA and 2WA the surgery. The mean difference is significant at P-value ≤ 0.05 level

GM^b^		1WB (pg/ml)^a^				1WA (pg/ml)^a^				2WA (pg/ml)^a^			
		Mean	Min-Max	*P*-valu^c^	*p*-value^d^	Mean	Min-Max	*P*-valu^c^	*p*-value^d^	Mean	Min-Max	*P*-valu^c^	*p*-value^d^
NGM	IL-10	498 ± 270	131–984		0.396	425 ± 232	93.5–867		0.147	459 ± 225	149–861		0.036
NI		378 ± 252	184–716	0.350		285 ± 196	121–523	0.225		203 ± 115	94.7–361	0.030	
RNI		271 ± 142	182–480	0.110		211 ± 101	93.2–337	0.079		265 ± 118	147–427	0.091	
NGM	IL-17	340 ± 193	92.1–711		0.145	448 ± 213	155–836		0.138	376 ± 171	78.7–645		0.687
NI		165 ± 54.8	94.8–228	0.995		299 ± 76.1	265–399	0.984		449 ± 107	306–561	0.307	
RNI		420 ± 221	159–632	0.377		585 ± 229	314–833	0.212		468 ± 249	190–725	0.389	

No statistically significant differences were seen for IL-17 across all time intervals. Nonetheless, the IL-17 levels in the NGM and RNI groups demonstrated an initial rise from 1WB to 1WA, subsequently followed by a decline in 2WA. In the NGM group, the average IL-17 levels rose from 340 ± 193 pg/ml in 1WB to 448 ± 213 pg/ml in 1WA, thereafter declining to 376 ± 171 pg/ml in 2WA. In the RNI group, IL-17 levels increased from 420 ± 221 pg/ml in 1WB to 585 ± 229 pg/ml in 1WA, thereafter declining to 468 ± 249 pg/ml in 2WA. Conversely, the NI group demonstrated a continuous elevation in IL-17 levels across the research duration, commencing at 165 ± 54.8 pg/ml in 1WB, escalating to 299 ± 76.1 pg/ml in 1WA, and culminating at 449 ± 107 pg/ml in 2WA, across the observed intervals.

**Fig. 2:**
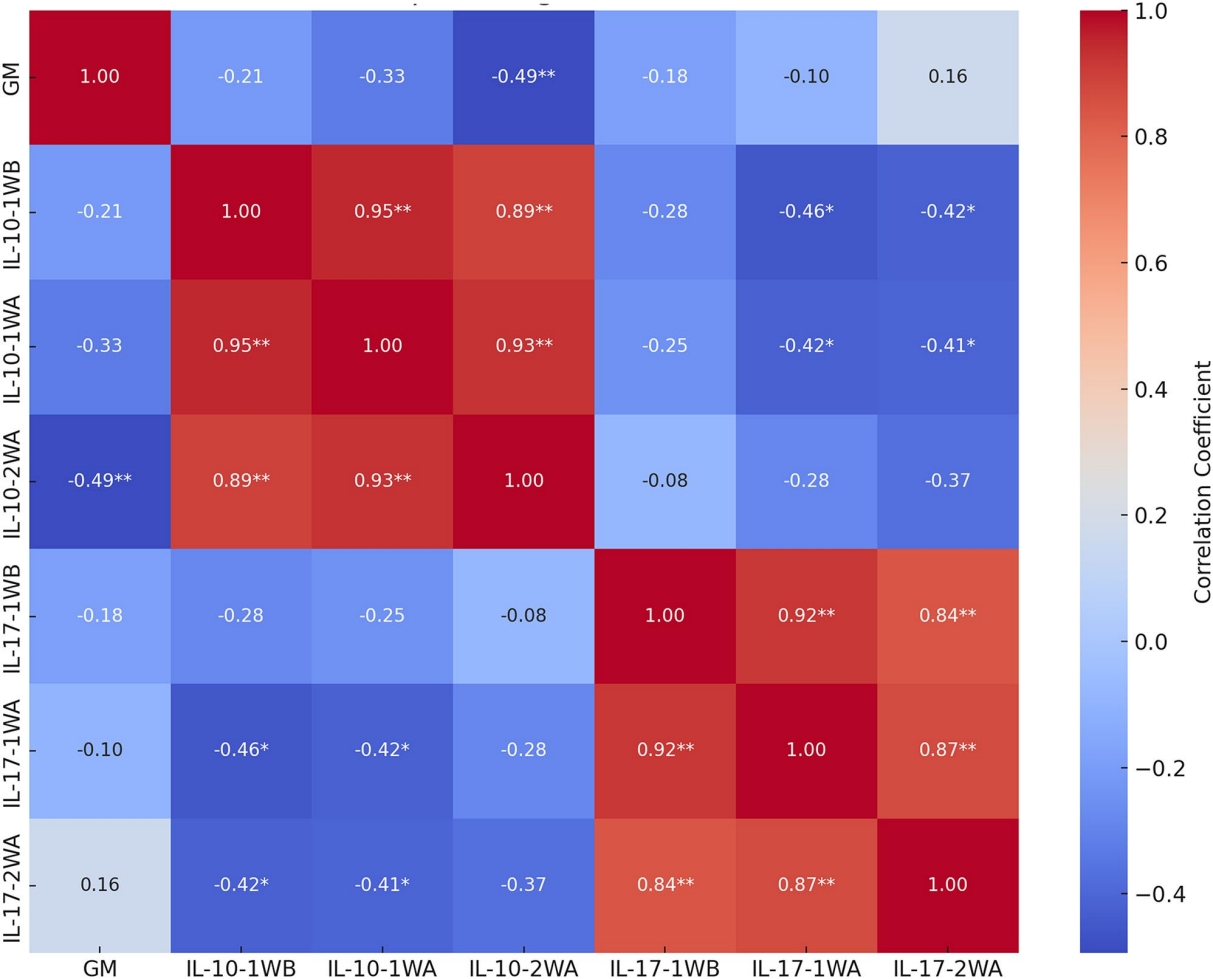
Heat map of Spearman’s rho analysis detects the correlations between Gut microbiota and cytokines (IL-10 and IL-17) at different time periods. ** Correlation is significant at the 0.01 level (2-tailed). * Correlation is significant at the 0.05 level (2-tailed)

Figure 2 depicts the correlations between particular immune cytokines (IL-10 and IL-17) at different time intervals and their relationship with gut microbiota (GM). IL-10-2WA demonstrates an inverse correlation with GM (-0.494, *p* < 0.01), suggesting that the gut microbiota may influence the production of anti-inflammatory cytokines. Moreover, IL-17 subtypes exhibit negative correlations with IL-10, particularly IL-17-1WB and IL-10-1WA (-0.419, *p* = 0.027), signifying an inverse relationship between pro-inflammatory and anti-inflammatory cytokines. These findings emphasize the complex interactions between immune responses and gut microbiota, underscoring the importance of these pathways in maintaining immune balance and their possible implications for inflammatory and autoimmune diseases.

## Discussion

The primary objective of this study was to elucidate the changes in gut microbiome (GM) composition in liver transplant (LT) patients and to investigate the potential associations between microbial dysbiosis, nosocomial infections (NIs), and immune response alterations post-transplantation. The results of this study demonstrate substantial shifts in the GM immediately following LT surgery, particularly in patients who developed NIs, alongside significant changes in immune markers such as interleukin (IL)-10 and IL-17. These findings underscore the intricate interplay between microbial ecology and immune function in LT patients’ population.

The gut microbiome of LT patients was profoundly altered across the peri-transplant period, with marked differences in the bacterial populations identified before and after the procedure. Prior to LT surgery, the GM was predominantly composed of species such as *Fusobacterium mortiferum*, *Enterococcus*, and *Ruminococcaceae*, taxa commonly associated with a healthy human gut microbiota. However, one week following LT, a significant shift in microbial composition was observed, characterized by an increase in the relative abundance of opportunistic pathogens, including *Klebsiella spp*., *Enterobacter spp*., and *Acinetobacter baumannii*, all of which are commonly implicated in hospital-acquired infections. This dysbiotic shift is likely multifactorial, driven by a combination of surgical intervention, the use of broad-spectrum antibiotics, and the immunosuppressive regimen required for graft survival [6, 26, 27]. These results corroborate findings from previous studies that suggest major surgical procedures, particularly organ transplants, can significantly disrupt the normal microbial balance, often leading to an increase in pathogenic organisms and a reduction in microbial diversity [6, 28]. Sucu et al. [29] reported an increase in Enterobacteriaceae and Enterococcaceae species, and a decrease in *Faecalibacterium prausnitzii* and Bacteroides in the overall variety of GM following LT. Similarly, Lai et al. [30] demonstrated that there is a change in microbial composition in LT patients as well as an unambiguous GM mark associated with the peri operative phase.

The observed overgrowth of *Klebsiella*,* Enterococcus*and *Enterobacter* species is particularly concerning, as these pathogens are frequently resistant to multiple classes of antibiotics and are major contributors to post-surgical infections in immunocompromised patients [31, 32]. The development of NIs in eight patients one week after LT, caused by pathogens such as *Escherichia coli* and *Klebsiella*, reinforces the LT recipients to infections, which can be exacerbated by hospital environments and dysbiosis. Notably, *Klebsiella spp*. are known for their ability to persist in the gut, complicating efforts to restore a healthy microbiome post-transplantation [33]. Interestingly, a probiotic bacterium *Bifidobacterium*, was isolated in some stool cultures after one week of surgery. *Bifidobacterium* inhabits the gut and counteract the growth of harmful bacteria. They also create a protective layer called a biofilm, inhibit the growth of pathogenic bacteria, decrease the presence of intestinal endotoxin and bacterial translocation, minimize cause damage to the intestinal mucosal epithelium, reduce its permeability, and slow down or prevent its degeneration [34–36]. A study conducted by Li et al. [37] and Ohland et al. [38] demonstrated that *Bifidobacterium* generates a significant amount of acidic compounds, leading to acidification of the intestinal cavity. This process accelerates the elimination of endotoxins and reduces harm to the intestinal mucosa. Consequently, it facilitates the rapid breakdown and absorption of nutrients in the gastrointestinal tract.

By contrast, after two weeks post-surgery, the GM composition showed partial recovery, with an increased presence of beneficial taxa such as *Akkermansia* and *Ruminococcaceae*. Both of these genera have been associated with improved gut barrier function and anti-inflammatory properties [39, 40]. However, the persistence of certain pathogens, such as *Klebsiella* and *Acinetobacter*, suggests that the recovery of the GM in LT patients is incomplete and may require prolonged intervention to fully restore microbial equilibrium.

The relationship between microbial dysbiosis and immune response alterations in LT patients was further examined by measuring cytokine levels, specifically IL-10 and IL-17, in patients at different period of time. IL-10 is a key anti-inflammatory cytokine, whereas IL-17 is a pro-inflammatory cytokine involved in the recruitment of immune cells to sites of infection [41, 42]. In patients who developed NIs post-LT, IL-10 levels decreased progressively from baseline (one week before LT) to one- and two-weeks post-transplantation, while IL-17 levels increased over the same period. These findings suggest a shift towards a pro-inflammatory immune environment in response to microbial dysbiosis and infection, which may exacerbate tissue damage and hinder recovery [43].

Interestingly, the changes in IL-10 levels were statistically significant in patients after two weeks of surgery, indicating that continuous infection may lead to chronic immune activation and an inability to mount an effective anti-inflammatory response. The decrease in IL-10 levels observed in these patients is consistent with the concept of immune exhaustion, where the immune system’s regulatory capacity is overwhelmed by the persistent inflammatory stimuli from ongoing infections [43]. The progressive rise in IL-17 levels, conversely, suggests that this cytokine may play a pivotal role in the inflammatory response to infection in LT patients, potentially contributing to the pathogenesis of graft dysfunction and the development of post-transplant complications [41].

### Advantages and limitations of the study

Limitations: The limited sample size of 31 liver transplant patients and 87 stool samples constitutes a primary limitation of our investigation. Furthermore, the statistical power of the analysis may be affected by the limited number of participants, despite the inclusion of patients at different time intervals (pre- and post-transplantation). The study is deficient in long-term follow-up, which would have offered a more thorough comprehension of the enduring consequences of GM changes on patient recovery and immunological function.

Advantages: Although these limitations, this study offers significant insights into the immune responses and gut microbiota dynamics in liver transplant patients, particularly concerning the emergence of nosocomial infections (NIs). The precise characterization of gut microbial changes at several time points (before and after liver transplantation) elucidates the impact of surgery, immunosuppression, and antibiotic treatments on gut microbial composition. The findings highlight significant microbial alterations potentially associated with infection development, which should guide future preventive efforts in post-transplant care. Moreover, the application of culture-based identification of GM and cytokine analysis enhances the understanding of the correlation between microbial dysbiosis and immune system changes, thereby laying a groundwork for future study in this domain.

## Conclusion

In summary, this study provides valuable insights into the alterations in gut microbiome composition and immune response following liver transplantation. The results demonstrate that microbial dysbiosis, characterized by an increase in opportunistic pathogens, is a common occurrence post-transplantation and is associated with the development of nosocomial infections and immune dysregulation. The observed shifts in IL-10 and IL-17 levels suggest that persistent infections may drive a pro-inflammatory immune response that complicates recovery and increases the risk of transplant-related complications. These findings highlight the need for comprehensive monitoring of both microbial and immune parameters in LT patients to improve clinical management and outcomes.

## Data Availability

All the data and materials used for the preparation of the manuscript are provided within the manuscript.

## Abbreviations

LT: Liver Transplantation
GM: Gut Microbiota
GIT: Human Gastrointestinal Tract
GEC: Gastroenterology Surgical Center
1WB: One Week Before LT
1WA: One Week After LT
2WA: Two Weeks After LT
ELISA: Enzyme-Linked Immunosorbent Assay
HCC: Hepatocellular Carcinoma
BCS: Budd-Chiari Syndrome
AIH: Autoimmune Hepatitis
LCF: Liver Cell Failure
NGM: Normal Gut Microbiota
NI: Nosocomial Infection
RNI: Recovered From Nosocomial Infection

## References

[1] Goel A, Gupta M, Aggarwal R. Gut microbiota and liver disease. Clin Gastroenterol Hepatol. 2014;29(6):1139–48. 10.1111/jgh.12556 24547986

[2] Zhang X-M, Fan H, Wu Q, Zhang X-X, Lang R, He Q. In-hospital mortality of liver transplantation and risk factors: a single-center experience. Annals Translational Med. 2021;9(5):369. 10.21037/atm-20-5618 PMC8033294 33842590

[3] Terrault NA, Francoz C, Berenguer M, Charlton M, Heimbach J. Liver transplantation 2023: status report, current and future challenges. Clin Gastroenterol Hepatol. 2023;21(8):2150–66. 37084928 10.1016/j.cgh.2023.04.005

[4] Zheng D, Liwinski T, Elinav E. Interaction between Microbiota and immunity in health and disease. Cell Res. 2020;30(6):492–506. 32433595 10.1038/s41422-020-0332-7 PMC7264227

[5] Ponziani FR, Valenza V, Nure E, Bianco G, Marrone G, Grieco A, Pompili M, Gasbarrini A. Effect of liver transplantation on intestinal permeability and correlation with infection episodes. PLoS ONE. 2020;15(6):e0235359. 32589654 10.1371/journal.pone.0235359 PMC7319319

[6] Medina C, Aykut B. Gut microbial dysbiosis and implications in solid organ transplantation. World J Transplantation. 2024;12(12):2792. 10.3390/biomedicines12122792 PMC11673786 39767699

[7] Sharma A, Giorgakis E. Gut microbiome dysbiosis in the setting of solid organ transplantation: what we have gleaned from human and animal studies. World J Transplantation. 2022;12(7):157. 10.5500/wjt.v12.i7.157 PMC9331413 36051453

[8] Rinninella E, Raoul P, Cintoni M, Franceschi F, Miggiano G, Gasbarrini A, Mele MC. What is the healthy gut microbiota composition? A changing ecosystem across age, environment, diet, and diseases. Microorganisms. 2019;7(1):14. 30634578 10.3390/microorganisms7010014 PMC6351938

[9] Chelala L, Kovacs CS, Taege AJ, Hanouneh IA. Common infectious complications of liver transplant. Cleve Clin J Med. 2015;82(11):773–84. 26540328 10.3949/ccjm.82a.14118

[10] Ferrarese A, Zanetto A, Becchetti C, Sciarrone S, Shalaby S, Germani G, Gambato, Martina R, Francesco P. Burra, P, Senzolo, M. Management of bacterial infection in the liver transplant candidate. World J Hepatol. 2018;10(2):222. 29527258 10.4254/wjh.v10.i2.222 PMC5838441

[11] Idossa D, Simonetto D. Infectious complications and malignancies arising after liver transplantation. Anesthesiol Clin. 2017;35(3):381–93. 28784215 10.1016/j.anclin.2017.04.002

[12] Zhong H, Liu C-Y, Dai Y-Q, Zhu C, Le K-J, Pang X-Y, Li Y-J, Gu Z-C, Yu Y-T. A bibliometric analysis of infectious diseases in patients with liver transplantation in the last decade. Annals Translational Med. 2021;9(22):1646. 10.21037/atm-21-2388 PMC8667120 34988155

[13] Boev C, Kiss E. Hospital-acquired infections: current trends and prevention. Crit Care Nurs Clin. 2017;29(1):51–65. 10.1016/j.cnc.2016.09.012 28160957

[14] Carlini V, Noonan D, Abdalalem E, Goletti D, Sansone C, Calabrone L, Albini A. The multifaceted nature of IL-10: regulation, role in immunological homeostasis and its relevance to cancer, COVID-19 and post-COVID conditions. Front Immunol. 2023;14:1161067. 37359549 10.3389/fimmu.2023.1161067 PMC10287165

[15] Kuwabara T, Ishikawa F, Kondo M, Kakiuchi TJM. The role of IL-17 and related cytokines in inflammatory autoimmune diseases. Mediat Inflamm. 2017;2017(1):3908061. 10.1155/2017/3908061 PMC5337858 28316374

[16] McGeachy M, Cua D, Gaffen S. The IL-17 family of cytokines in health and disease. Immunity. 2019;50(4):892–906. 30995505 10.1016/j.immuni.2019.03.021 PMC6474359

[17] Zenobia C, Hajishengallis G. Basic biology and role of interleukin-17 in immunity and inflammation. Periodontol 2000. 2015;69(1):142–59. 26252407 10.1111/prd.12083 PMC4530463

[18] Niu J, Yue W, Song Y, Zhang Y, Qi X, Wang Z, Liu B, Shen H, Hu X. Prevention of acute liver allograft rejection by IL-10-engineered mesenchymal stem cells. Clin Exp Immunol. 2014;176(3):473–84. 24527865 10.1111/cei.12283 PMC4008992

[19] Oh P, Martinez I, Sun Y, Walter J, Peterson D, Mercer D. Characterization of the ileal microbiota in rejecting and nonrejecting recipients of small bowel transplants. Am J Transplant. 2012;12(3):753–62. 22152019 10.1111/j.1600-6143.2011.03860.x

[20] Pirozzolo I, Li Z, Sepulveda M, Alegre M-L, Transplantation L. Influence of the microbiome on solid organ transplant survival. J Heart lung Transplantation. 2021;40(8):745–53. 10.1016/j.healun.2021.04.004 PMC8319047 34030971

[21] Amies C. A modified formula for the preparation of Stuart’s transport medium. Can J Public Health/Revue Canadienne De Sante’e Publique. 1967;58(7):296–300. 4859908

[22] Levett P, Murray P, Baron E, Jorgensen J, Landry M, Pfaller M. Manual of clinical microbiology. UK: Oxford University Press; 2006.

[23] Ellner P, Stoessel C, Drakeford E, Vasi F. New Culture Medium for Medical Bacteriology. Am J Clin Pathol. 1966;45(4):502–4. 5325709 10.1093/ajcp/45.4_ts.502

[24] Downes F, Ito K. Compendium of methods for the microbiological examintion of foods–APHA. Washington, DC Ed. 2001;4.

[25] Chan D, Perlstein N, Immunology. A practical Guide Eds. Academic: New York.; 1987. p. p71.

[26] Francino M. Antibiotics and the human gut microbiome: dysbioses and accumulation of resistances. Front Immunol. 2016;6:164577. 10.3389/fmicb.2015.01543 PMC4709861 26793178

[27] Swarte J, Li Y, Hu S, Björk J, Gacesa R, Vich Vila A, Douwes R, Collij V, Kurilshikov A. Gut microbiome dysbiosis is associated with increased mortality after solid organ transplantation. Sci Transl Med. 2022;14(660):7566. 10.1126/scitranslmed.abn7566 36044594

[28] Tsigalou C, Paraschaki A, Bragazzi N, Aftzoglou K, Stavropoulou E, Tsakris Z, Vradelis S, Bezirtzoglou E. Alterations of gut microbiome following gastrointestinal surgical procedures and their potential complications. Front Cell Infect Microbiol. 2023;13:1191126. 37333847 10.3389/fcimb.2023.1191126 PMC10272562

[29] Sucu S, Basarir K, Mihaylov P, Balik E, Lee J, Fridell J, Emamaullee J, Ekser B. Impact of gut microbiota on liver transplantation. Am J Transplant. 2023;23(10):1485–95. 37277064 10.1016/j.ajt.2023.05.030

[30] Lai Z, Chen Z, Zhang A, Niu Z, Cheng M, Huo C, Xu J. The gut microbiota in liver transplantation recipients during the perioperative period. Front Physiol. 2022;13:854017. 35530507 10.3389/fphys.2022.854017 PMC9075733

[31] Salimiyan R, Ghazvini K, Farsiani H. Clinical and pathogenesis overview of Enterobacter infections. Clin Med. 2020;6(4):146–54.

[32] Martin R, Bachman M. Colonization, infection, and the accessory genome of Klebsiella pneumoniae. Front Cell Infect Microbiol. 2018;8:4. 29404282 10.3389/fcimb.2018.00004 PMC5786545

[33] Paczosa M, Mecsas J. reviews mb. Klebsiella pneumoniae: going on the offense with a strong defense. Microbiology and Molecular biology reviews. 2016;80(3):629 – 61. 10.1128/MMBR.00078-15 PMC4981674 27307579

[34] Holma R, Kekkonen R, Hatakka K, Poussa T, Vapaatalo H, Adlercreutz H, Korpela R. Low serum enterolactone concentration is associated with low colonic Lactobacillus–Enterococcus counts in men but is not affected by a synbiotic mixture in a randomised, placebo-controlled, double-blind, cross-over intervention study. Br J Nutr. 2014;111(2):301–9. 23919920 10.1017/S0007114513002420

[35] Baek S, Kim S, Lee C, Roh K, Keum B, Kim C, Kim J. Relationship between the severity of diversion colitis and the composition of colonic bacteria: a prospective study. Gut Liver. 2014;8(2):170. 24672659 10.5009/gnl.2014.8.2.170 PMC3964268

[36] Zhu D, Chen X, Wu J, Ju Y, Feng J, Lu G, Ouyang M, Ren B, Li Y. Effect of perioperative intestinal probiotics on intestinal flora and immune function in patients with colorectal cancer. J South Med Univ. 2012;32(8):1190–3. 22931620

[37] Li Y, Chen Y, Zhang J, Zhu J, Liu Z, Liang S, Sun K, Liao W, Gong J. Protective effect of glutamine-enriched early enteral nutrition on intestinal mucosal barrier injury after liver transplantation in rats. Am J Surg. 2010;199(1):35–42. 20103064 10.1016/j.amjsurg.2008.11.039

[38] Ohland C, MacNaughton W. Probiotic bacteria and intestinal epithelial barrier function. Am J physiology-gastrointestinal Liver Physiol. 2010;298(6):G807–19. 10.1152/ajpgi.00243.2009 20299599

[39] Liu R, Rowan-Nash A, Sheehan A, Walsh R, Sanzari C, Korry B, Belenky P. Reductions in anti-inflammatory gut bacteria are associated with depression in a sample of young adults. Brain Behav Immun. 2020;88:308–24. 32229219 10.1016/j.bbi.2020.03.026 PMC7415740

[40] Pellegrino A, Coppola G, Santopaolo F, Gasbarrini A, Ponziani F. Role of Akkermansia in human diseases: from causation to therapeutic properties. Nutrients. 2023;15(8):1815. 37111034 10.3390/nu15081815 PMC10142179

[41] Jin W, Dong C. infections. IL-17 cytokines in immunity and inflammation. Emerging microbes & infections. 2013;2(1):1–5. 10.1038/emi.2013.58 PMC3820987 26038490

[42] Iyer S, Cheng G. Role of interleukin 10 transcriptional regulation in inflammation and autoimmune disease. Crit Rev Immunol. 2012;32(1):23–63. 22428854 10.1615/critrevimmunol.v32.i1.30 PMC3410706

[43] Gao Z, Feng Y, Xu J, Liang J. T-cell exhaustion in immune-mediated inflammatory diseases: new implications for immunotherapy. Front Immunol. 2022;13:977394. 36211414 10.3389/fimmu.2022.977394 PMC9538155

